# Acute exercise response in the critically ill

**DOI:** 10.1186/cc12477

**Published:** 2013-03-19

**Authors:** N Collings, R Young

**Affiliations:** 1 University Hospital Southampton, UK; 2 Sheffield Hallam University, Sheffield, UK

## Introduction

This study aims to quantify the acute exercise response to early passive and active activities in order to inform exercise prescription when designing rehabilitation programmes for the critically ill. Critical care survival is often associated with a poor functional outcome [1], with recent investigations presenting the case for early rehabilitation in order to optimise functional recovery [2]. There, remains, however, a scarcity of research investigating the immediate response to exercise and subsequent exercise prescription, in the acute phase following critical illness.

## Methods

This study is a prospective randomised controlled trial with a repeated-measures crossover design. Eligible participants, requiring mechanical ventilation for 4 or more days, completed two exercise activities routinely used in early critical care rehabilitation, a passive chair transfer (PCT) and active sitting on the edge of the bed (SOEOB). The oxygen consumption and cardiovascular parameters were measured to quantify and compare the exercise response between the two activities.

## Results

Data are presented as the median (interquartile range). Data for five patients have been collected, aged 68 years (23), with an ITU stay of 15 days (10.5) and duration of mechanical ventilation 8 days (12), at the point of intervention. Exercise response results are reported (Table 1).

**Table 1:** 

Parameter	PCT (*n *= 5)	SOEOB (*n *= 5)
Oxygen consumption (ml/minute)	270 (46.5)	334 (121)
Carbon dioxide production (ml/minute)	142 (45.5)	185 (121)
Heart rate (bpm)	92 (24)	77.5 (29)
Mean arterial pressure (mmHg)	100.3 (20)	97.3 (13)
Minute ventilation (l/minute)	11.0 (3.5)	12.1 (3.4)

## Conclusion

Intensive care patients with prolonged mechanical ventilation demonstrate a higher rate of oxygen consumption when actively sitting on the edge of the bed, compared with a passive chair transfer. This may have important consequences for early mobilisation of the critically ill.
